# Combined Periodontal and Endodontic Management of Palatal Radicular Groove with Platelet-Rich Fibrin and Biodentine®

**DOI:** 10.1155/2022/6461654

**Published:** 2022-11-09

**Authors:** Arjun Hari Rijal, Bhageshwar Dhami, Pratistha Ghimire

**Affiliations:** ^1^Department of Periodontology and Oral Implantology, Kathmandu University School of Medical Sciences, Dhulikhel, Kavrepalanchok, Nepal; ^2^Department of Periodontology and Oral Implantology, Kantipur Dental College and Hospital, Basundhara, Kathmandu, Nepal; ^3^Dental Department, Methinkot Hospital, Kavrepalanchok, Nepal

## Abstract

Palatal radicular groove (PRG) is developmental anomaly of root, which when present is associated with periodontal and endodontic problems. Different treatment modalities are available for its management, such as periodontal flap surgery, endodontic management, sealing of PRG with various cements, bone graft procedure for intrabony defect, extraction with intentional replantation after sealing or removal of a PRG, and endodontic treatment as well as the use of various growth factors. Success of the therapy depends on the type, depth, and extent of PRG, presence or absence of intrabony defect, vitality of involved tooth, and types of materials used to seal PRG. This study reports management of PRG with Biodentine® and platelet-rich fibrin in a 44-year-old systemically healthy female patient.

## 1. Introduction

Different morphological variations associated with maxillary anteriors include congenital absence of tooth, supernumerary tooth, dens invaginatus, Eagle's talon, peg-shaped lateral incisor, gemination, fusion, accessory root, and palatal gingival grooved incisors [1, 2]. Palatal radicular groove (PRG) is a developmental anomaly usually on the palatal surface of maxillary lateral incisors either mesially or distally on the root. It was first described as radicular groove by Black [3], whereas the term ‘palatogingival groove' was first coined by Lee et al. [4]. Several technical terms have been proposed to describe the PRG, such as distolingual groove, corono-radicular groove, radicular lingual groove, vertical developmental radicular groove, cingulo-radicular groove, developmental radicular anomaly, interruption groove, PRG, and palatoradicular groove [4–7]. Various hypothesis proposed for the development of PRG include failed attempt to form new root [5, 8], infoldings of Hertwigs epithelial root sheath and inner enamel epithelium [4], or change in the genetic mechanism [9]. Most commonly followed classification for PRG is based on the extent of groove on the surface of root, which was given by Gu [10].

Type I: the groove is short (not beyond the coronal third of the root).

Type II: the groove is long (beyond the coronal third of the root) but shallow, corresponding to a normal or simple root canal.

Type III: the groove is long (beyond the coronal third of the root) and deep, corresponding to a complex root canal system.

Entrance of PRG acts as a site for plaque accumulation, leading to gingivitis and bleeding on probing. In advance cases, PRG allows passage of microorganisms throughout its length, leading to the loss of periodontal attachment and intrabony defects. This is presented as a combined type of primary periodontal lesion with secondary involvement of the pulp. The periodontal breakdown and root surface contamination result in retrogenic pulp necrosis even though PRGs do not reach the apex and are not very deep. Accessory foramina or dentinal tubules along the grooves have been regarded as the possible pathway between the pulp canal and the groove [11, 12].

Different treatment modalities available for the management of PRG include simple gingival curettage of inflamed gingival tissues, saucerization of shallow groove with diamond bur, root canal therapy of involved tooth, and various surgical procedures (periodontal flap with sealing of groove with cement, guided tissue/bone regeneration if intrabony defect present, intentional root canal therapy, and reimplantation). In this study, we have reported successful management of primary periodontal secondary endodontic lesion complicated by class II PRG with Biodentine® and platelet-rich fibrin (PRF).

## 2. Case Presentation

**Figure 1:**
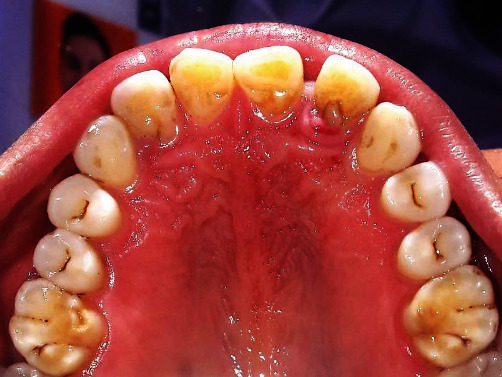
Palatal radicular groove with reference to 22.

**Figure 2:**
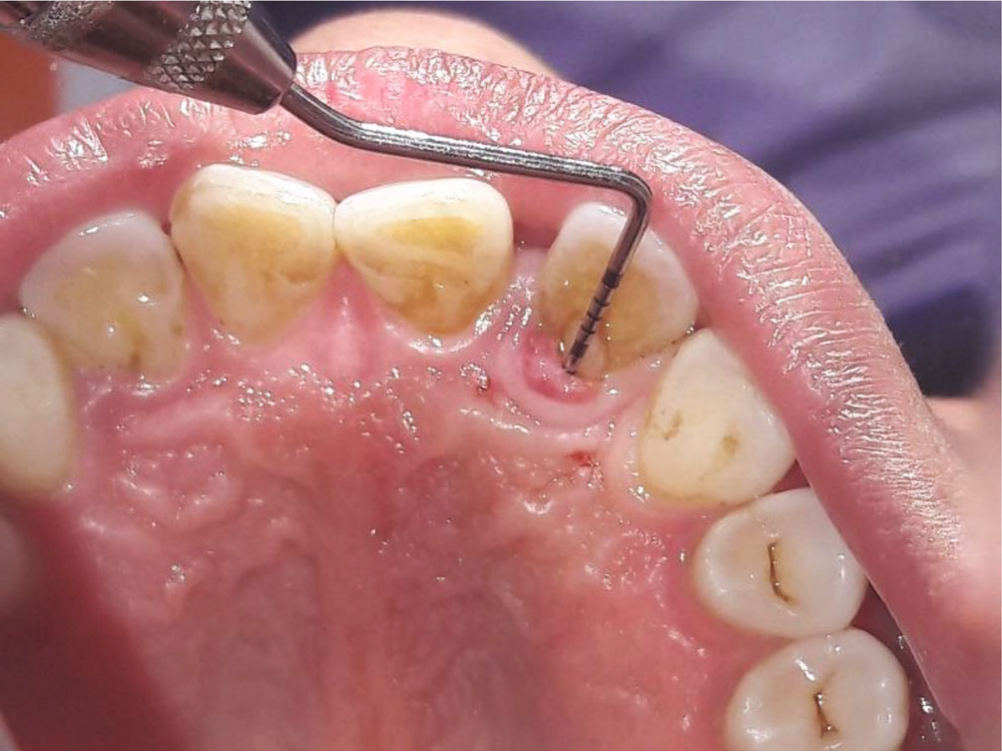
Preoperative pocket probing depth of 10 mm with reference to 22.

**Figure 3:**
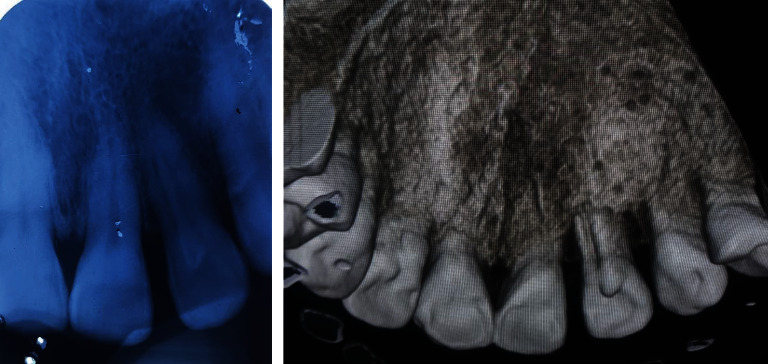
Preoperative periapical radiograph and cone-beam computed tomography.

A 44-year-old female visited the Department of Periodontology and Oral Implantology, Kantipur Dental College Teaching Hospital and Research Centre, Basundhara, Kathmandu, Nepal, with a chief complaint of pain and swelling of gingiva in upper left front region of the jaw for one month. The pain was mild, dull, and continuous in nature. There was no history of trauma, swelling, and pain with respect to the concerned tooth. General examination revealed that the patient was moderately built and nourished. Clinical examination revealed intact crown with no carries or fracture. Vitality testing was negative, whereas positive response was elicited to vertical percussion. There was presence of localized circumscribed gingival inflammation on palatal aspect with respect to the mesio-palatal line angle of 22 (i.e., maxillary left lateral incisor) with pocket probing depth of 10 mm, whereas there was only 3 mm of pocket probing depth on labial aspect of the involved tooth (Figures 1 and 2). Intraoral periapical radiograph of the tooth showed a periapical radiolucency as well as a radiopaque line extending from cingulum of crown to the middle third of the root. No intrabony vertical defect was noted. Cone-beam computed tomography (CBCT) revealed extension of groove up to the middle third of root with interdental bone loss (Figure 3).

Based on these clinical and radiographic examination and findings, it was diagnosed to be the primary periodontal and secondary endodontic lesions.

**Figure 4:**
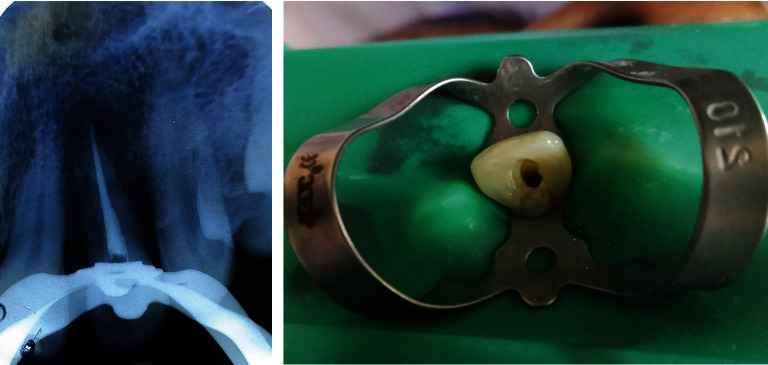
Endodontic management of involved tooth before surgery.

**Figure 5:**
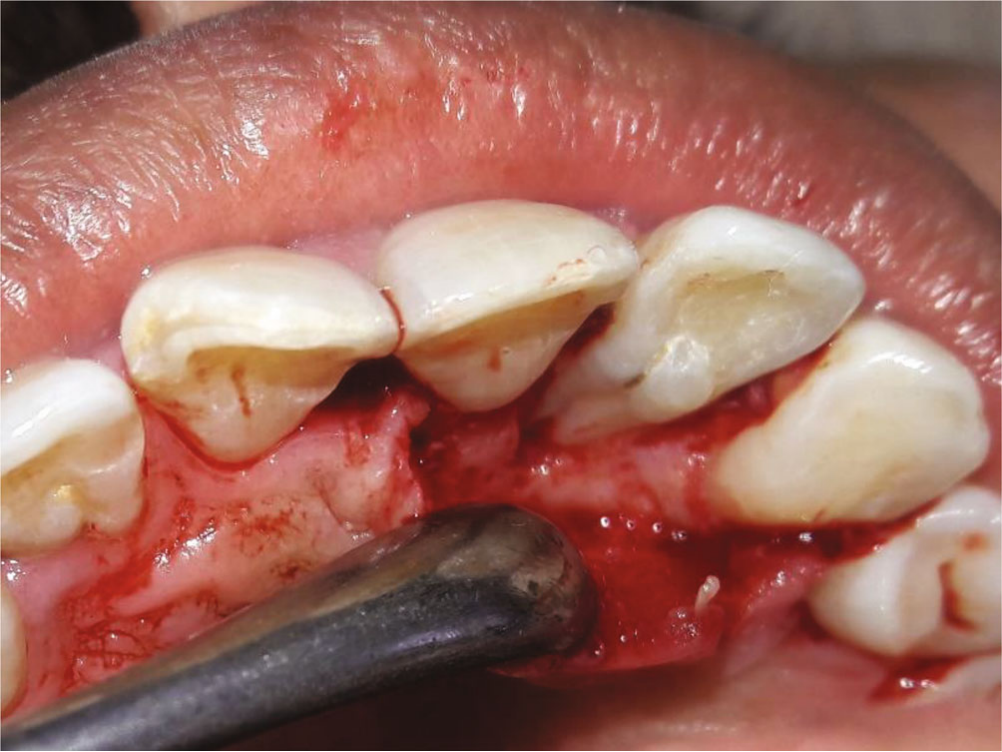
Flap reflection and debridement of the defect.

**Figure 6:**
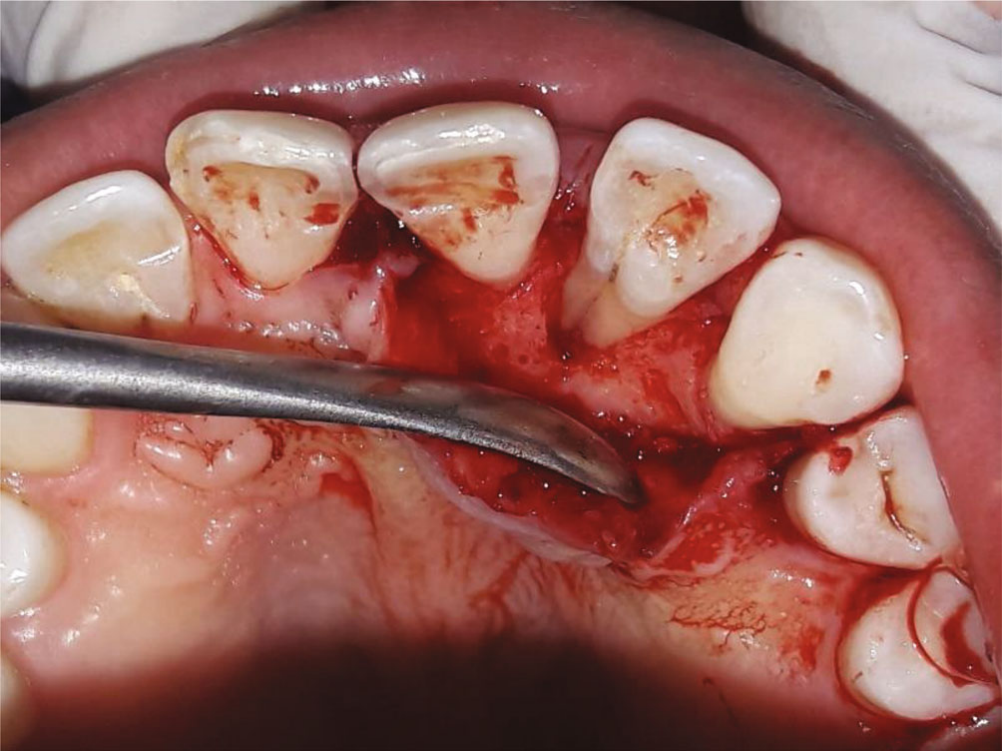
Root conditioning with 24% EDTA.

**Figure 7:**
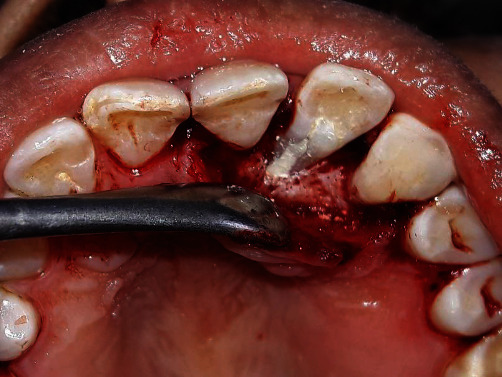
Sealing of groove with Biodentin.

**Figure 8:**
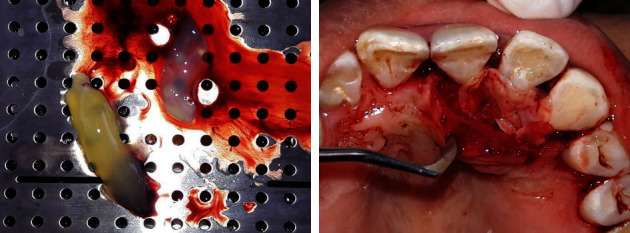
Preparation and placement of platelet-rich fibrin.

**Figure 9:**
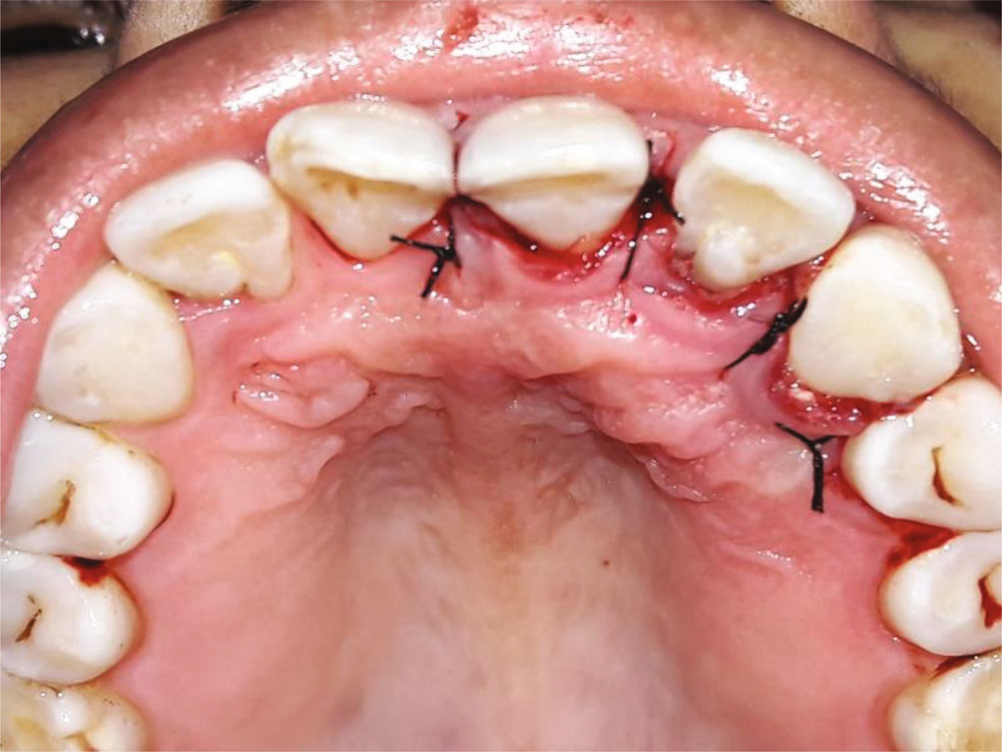
Simple interrupted suturing with 4-0 silk suture.

For the management of the case, non-surgical periodontal therapy (scaling and root planning) was done initially. Patient was referred to the endodontist for root canal treatment, and root canal therapy was completed (Figure 4). One month after completion of endodontic therapy, sealing of PRG with Biodentine® and PRF was planned. Prior to surgery, mouth rinse with 0.2% chlorohexidine gluconate for one minute was advised to the patient. Adequate anaesthesia was achieved with local infiltration of 2% lidocaine with adrenaline in 1 : 200,000 concentration. Full thickness envelope flap was raised on palatal aspect (Figure 5). Complete debridement was done with removal of granulation tissue. The PRG was smoothened with diamond bur under copious sterile saline irrigation. Conditioning of root was done with 24% ethylenediaminetetraacetic acid (EDTA) for two minutes and removed with sufficient amount of sterile saline solution (Figure 6). Then, the PRG was sealed with Biodentine® (Biodentine® Bioactive Dentin Substitute Septodont; Figure 7). Intravenous blood (by venipuncturing of the antecubital vein) was collected in a 10 ml sterile glass tube (BD Vacutainer® blood collection tubes, 10 ml) without anticoagulant and immediately centrifuged (IntraSpin® system by INTRA-LOCK, Biodentine: Septodont North America Medical Equipment ManufacturingLancaster, PABDVacutainer: JIANGSU HXRT MD CO., LTD. Jiangyan District, 225500, Taizhou City, Jiangsu Province, ChinaIntraspin: Biohorizon Birmingham, AL 35244 USA) at 3,000 rpm for 10 minutes. PRF was separated from red corpuscles base (preserving a small RBC layer) using sterile tweezers just after removal of platelet-poor plasma and then transferred into a sterile dappen dish. PRF was squeezed between two gauze pieces to remove excess of plasma. PRF was placed above the root surface (Figure 8). Finally, flap was approximated with 4-0 silk suture (ETHILON®, Ethicon Inc., Johnson & Johnson, Piscataway, NJ, USA) using simple interrupted suturing technique (Figure 9).

**Figure 10:**
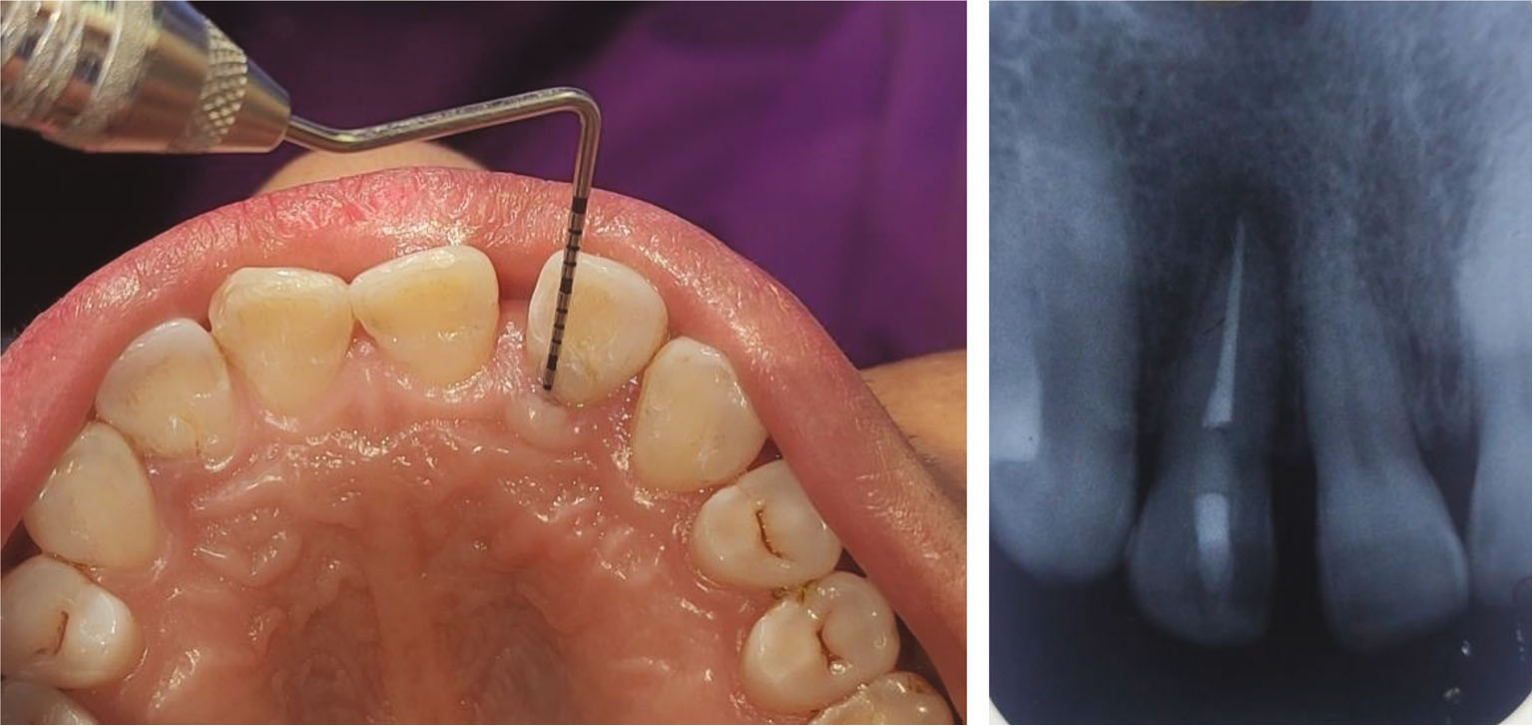
Postoperative six months follow-up with pocket probing depth of 4 mm with reference to 22.

**Figure 11:**
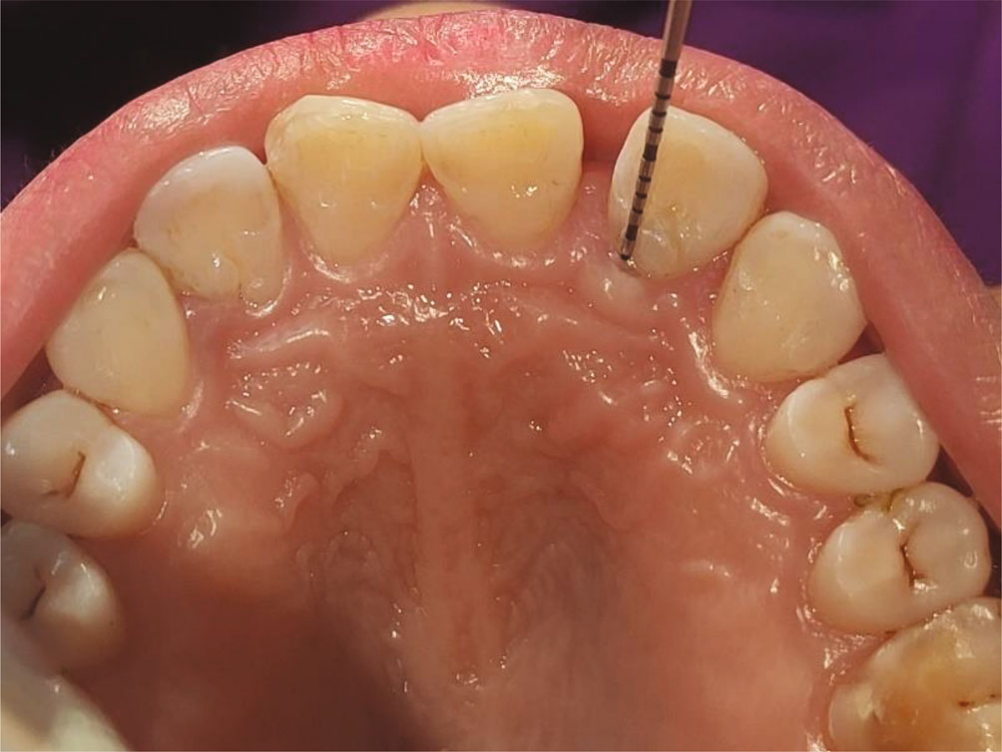
Postoperative one year follow-up with pocket probing depth of 4 mm with reference to 22.

**Figure 12:**
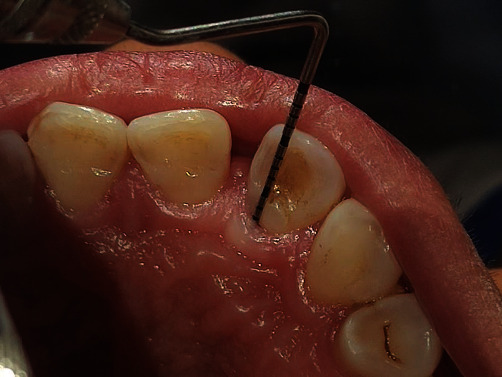
Postoperative two years follow-up with pocket probing depth of 3 mm with reference to 22.

**Figure 13:**
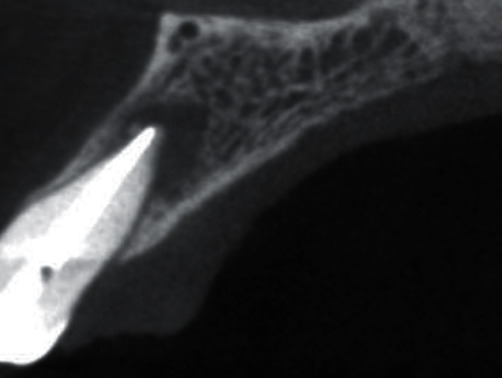
Postoperative CBCT showing healing of lesion on the labial aspect.

**Figure 14:**
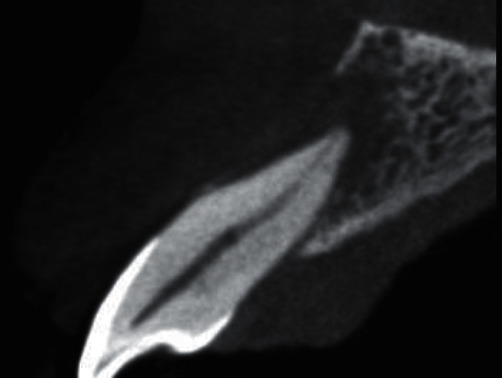
Preoperative CBCT showing extensive loss of bone on the labial aspect.

Postsurgically, the surgical area was covered with periodontal dressing (COE-PAK™, (De Trey/Denstply, Konstanz, Germany) Automix Surgical Dressing & Periodontal Pack). Postsurgical instructions were given to the patient. Non-steroidal anti-inflammatory drugs (NSAIDS) (Ibuprofen 400 mg and Paracetamol 375 mg, Flexon Tablet, Aristo Pharmaceuticals Pvt. Ltd.), (23-A, Shah Industrial Estate, Off Veera Desai Road, Andheri (West), Mumbai – 400 053, India.) three times daily for three days) for inflammation control and pain relief and 0.2% chlorhexidine gluconate (CHX oral rinse—100 ml mouth wash) two times a day for 2 weeks were prescribed. After two weeks of surgery, periodontal dressing was removed, and gingival healing was found to be satisfactory and uneventful. Patient was kept on regular follow-up (Figures 10 and 11). Complete examination after 2-year follow-up showed reduction in PPD to 3 mm, and tooth was asymptomatic endodontically though there was presence of periapical lesion, which might be because of healing by scar formation (Figures 12 and 13). In addition, there was reduction in size of radiolucency as compared to preoperative CBCT image (Figures 13 and 14).

## 3. Discussion

PRG is morphological as well as developmental anomaly, which begins from or near cingulum and extend along the various length of root of maxillary anteriors. There is wide variation in prevalence of PRG. The variation in prevalence may be due to difference in diagnostic criteria or examination methodologies used, for example, evaluation of extracted teeth versus clinical examination, and clinical examination versus CBCT study (Table 1).

Diagnosis of PRG is very difficult because of its morphological similarity with other anomalies like dens invaginatus, Tomes' root, and extra root variation. In addition, history provided by the patients are non-specific that may resemble with other clinical conditions (e.g., primary or secondary endodontic lesion). Therefore, diagnosis of PRG is mainly done by taking proper patient history, clinical examination, and advanced radiography (CBCT) [21]. Patient usually complaints of continuous dull or acute pain, gingival swelling, and mobility of teeth, which is not associated with dental caries, trauma, or discoloration of the teeth, whereas in some cases, patient may be asymptomatic. In the current case, the patient presented with dull continuous pain along with swelling of gingiva.

Clinical examination may reveal funnel shaped hollow grooves with an accumulation of plaque and calculus, increased pocket probing depth, loss of epithelial attachment, swollen gingiva, and bleeding on probing [21]. Diagnosis of either periodontal or primary endodontic lesion can be made by electric pulp testing, heat test, or cold test. Advanced cases with deep groove, loss of attachment, and increased pocket do not respond to electric or thermal pulp testing [22, 23]. Intraoral periapical radiographs can be used to diagnose PRG associated with periodontal or endodontic lesion. Widening of periodontal ligament space can be seen in primary periodontal lesion, whereas tear-drop shaped radiolucency may be observed in the case of primary endodontic infection [5]. Due to superimposition of various structures, it is difficult to diagnose PRG with two-dimensional radiographs [6]. Therefore, to avoid such difficulty and for proper diagnosis, CBCT was advised in the current case. In this case, increase in pocket probing depth, attachment loss, periapical radiolucency, and para-pulpal line signifies primary periodontal infection with secondary endodontic lesion complicated by PRG. Other differential diagnoses, such as vertical root fracture, gemination, and fused roots, were ruled out based on the clinical examination and CBCT findings.

Previously, if patient had presented with clinical findings of PRG along with draining sinus and advanced periodontitis, treatment of choice would be extraction because of poor prognosis [5]. However, with the development of advanced diagnostic tool, better sealing material, and regenerative material, different treatment options are available for patient with periodontal and endodontic infection complicated by PRG. Basic principles for the management of PRG are complete removal of microorganism, sealing of groove with cement to prevent communication between root canal and periodontium and periodontal regeneration, and complete healing of the periodontium [21]. If the dental pulp is involved, endodontic treatment is recommended before the periodontal approach.

Different treatment modalities, such as periodontal flap surgery, endodontic management, sealing of PRG with various cements, bone graft procedure for intrabony defect, extraction with intentional replantation after sealing or removal of a PRG, and endodontic treatment, and various growth factors are available for the management. In case of mild PRG, odontoplasty in conjunction with periodontal treatment like curettage and root planning and saucerization of groove with diamond round bur can be performed [24]. Similarly, in complex PRG with advanced periodontal infection, various treatment options have been proposed, which includes removal of granulation tissue with periodontal flap, saucerization of groove and sealing with different materials, guided tissue regeneration with or without bone graft, intentional reimplantation, and extraction [25].

Several sealing materials including amalgam, glass ionomer cement, composite resin, calcium silicate-based cement like mineral trioxide aggregate, and Biodentine® have been used to seal moderate-to-deep PRG [21].

Biodentine® is tricalcium-based cement, which is available as powder and liquid. Components of powder are tricalcium silicate, dicalcium silicate, calcium carbonate and oxide, iron oxide, and zirconium oxide. Components of liquid are calcium chloride and water-soluble polymer. Biodentine® has several advantages like easy manipulation during mixing and sealing, short setting time of 9–12 minutes, improved mechanical properties, good biocompatibility, and regenerative potential [2, 26, 27]. In addition, tricalcium silicate-based cements are considered as the best sealing material for grooves since it does not leach any contaminants [27]. Because of significant advantages of Biodentine®, we used this material to seal moderate type of PRG.

In cases of complex PRG with advanced periodontal infection, various regenerative processes and materials are required for successful management. Guided tissue/bone regeneration with membranes, bone grafts, PRF [27], and enamel matrix proteins [28, 29] has been used for successful management of complex cases. PRF is autologous material, which have various growth factors that help in various regenerative procedures. PRF consists of a fibrin matrix polymerized in a tetra molecular structure, with the incorporation of platelets, leukocyte, and cytokines, and circulating stem cells [30]. Growth factors present in PRF are transforming growth factor type beta 1, platelet-derived growth factor-AB, vascular endothelium growth factor , and glycoproteins (such as thrombospondin-1) [31]. PRF contains cytokines, and cells are entrapped in a matrix, which is released after a short period and can serve as a resorbable membrane. The rationale behind the use of PRF membrane is that the platelet *α*-granules are a reservoir of many growth factors that are known to play a definitive role in tissue repair [30].

Johns et al. [27] reported successful management of PRG with advanced periodontal infection with root canal therapy, apicectomy, sealing of groove with Biodentin, and bone defect management with bone graft and PRF membrane. After two years, there was significant reduction in PPD, patient was asymptomatic with a 3 mm non-bleeding sulcus, and there was reduction in size of radiolucency radiographically. Dhanyakumar and Devasia [32] treated PRG with combined endodontic–periodontal approach, and Biodentine® was used to seal PRG. Patient was asymptomatic, and significant reduction in PPD and size of radiolucency was noted after three months. Our case reports confirmed that PRF alone could be successfully used for the treatment of PRG-related periodontal infection and Biodentine® to seal the shallow-to-moderate PRG, when an adequate primary closure was obtained.

## 4. Conclusion

This case report described a management technique that resulted in clinical and radiographic resolutions of PRG-related periodontal attachment loss by a periodontal regenerative approach with PRF. Also, sealing of PRG with Biodentine® which is a biocompatible material eliminates the risk of adverse tissue response. Biodentine® also has outstanding sealing properties that reduces the risk of clinical failures through bacterial percolation. Biodentine® needs no surface conditioning or bonding and sets quickly, making it simple and easy to use. Currently, no other major disadvantage of the use of PRF has been reported. Therefore, combination of these materials was chosen to treat the complications that would arise due to the presence of PRG. Early diagnosis and suitable treatment plan of PRG-related defect can modify the prognosis and successful treatment of involved tooth.

## Figures and Tables

**Table 1:** Prevalence of palatal radicular groove.

S.No.	References	Evaluation methods	Prevalence
1	Everett and Kramer [6]	Survey of 625 extracted maxillary lateral incisors	<2%
2	Withers et al. [13]	Clinical examination of 531 maxillary incisor	Overall 8.5%
		Total 2,099 maxillary incisor examined	2.33%
			Maximum 93.8% in maxillary lateral incisors
3	Kogon [7]	3,168 extracted maxillary incisors	Overall 4.6%
			Central incisors 5.6%
			Lateral incisors 3.4%
4	Storrer et al. [14]	73 extracted maxillary lateral incisors	9.58%
5	Al-Rasheed [15]	Clinical examinations of 552 maxillary lateral incisors in 276 Saudi adults	10.3%
6	Hou and Tsai [16]	Clinical examinations of 404 maxillary incisors in 101 individuals	18.1%
7	Shreshta et al. [17]	Clinical examination of 1,362 maxillary anterior teeth	0.88% for lateral incisors
8	Khan et al. [18]	250 patients examined	7.6% of central incisors and 13.4% of lateral incisors
9	Neves et al. [19]	1,668 extracted maxillary central incisors	1.61%
10	Aksoy et al. [20]	CBCT examinations of 993 teeth (330 canines, 315 lateral incisors, and 348 central incisors)	0.57% of central incisors and 2.22% of lateral incisors

## Data Availability

Data supporting this research article are available from the corresponding author or first author on reasonable request.
